# In vitro comparative quality evaluation of different brands of Amlodipine Tablets Commercially available in Jimma Town, South-western Ethiopia

**DOI:** 10.1371/journal.pone.0310828

**Published:** 2024-11-19

**Authors:** Abera Milkesa, Gemmechu Hasen, Tesfaye Mohammed, Yesuneh Tefera Mekasha, Duresa Dedefo, Belachew Umeta, Sultan Suleman

**Affiliations:** 1 Jimma Medical Center, Hospital Pharmacy Unit, Jimma, Oromia, Ethiopia; 2 School of Pharmacy and Laboratory of Drug Quality (JuLaDQ), Jimma University, Jimma, Oromia, Ethiopia; 3 Pharmaceutical Sciences, Pharmaceutical Quality Assurance and Regulatory Affairs, University of Gondar, Gondar, Ethiopia; 4 Department of Pharmacy, Southern Nations, Nationalities and Peoples, Hossana College of Health Science, Hossana, Ethiopia; University of Agriculture Faisalabad, PAKISTAN

## Abstract

**Background:**

The incidence of hypertension in persons 25 years of age and older is estimated to be 46% in Africa, where it is still very common. This concerning rate could be explained by the pharmaceutical markets’ accessibility to poor quality antihypertensive drugs. Thus, the purpose of this study was to evaluate and compare the quality different brands of Amlodipine Tablets Commercially available in Jimma Town, South-western Ethiopia.

**Methods:**

The quality control test was conducted from August 30, 2019 to February 27, 2020 at Jimma University in the Laboratory of Drug Quality Control (JuLaDQ). The laboratory test was carried out in accordance with WHO inspection guidelines and United States Pharmacopeia. A statistically significant was considered when P<0.05. For further comparison of the *in-vitro* dissolution profiles of amlodipine tablets, model-independent model-dependent parameters and statistical Dunnetts tests for ensuring bioequivalence were used to further compare the *in-vitro* dissolution profiles.

**Results:**

With the exception of brand AMD-5 (1/10), the remaining nine (n = 9) brands were within WHO visual inspection criteria. The quality control parameters such as friability, weight variation, identity, assay, and dissolution test were within the United States Pharmacopeia. The model independent parameters (f1, and f2) confirmed that, all generic products were bio-equivalence, and interchangeable with comparator product. The model dependent approaches revealed the Weibull model (AMD-10), the Zero order (AMD-3), and the Korsemeyer-Peppas models were the most effective predictions for the release of the pharmaceutical substance from the dosage form. The Korsemeyer-Peppas model (r2 ≥0.9695) was the best descriptive model for determining the amlodipine drug kinetics from the point of view of all brands examined. The evaluated amlodipine brand tablets were in line with quality standards. The model independent methods confirmed that the generic brand tablets were interchangeable in clinical practice. The tested products follow more than two drug release kinetics.

**Conclusion:**

The study revealed a manifest discrepancy in the dissolution profiles’ releases. Therefore, it is strongly advised to use appropriately designed dissolution profile evaluation methods with various pH values in the dissolution media, as well as to do comprehensive visual inspections. This will make it easier to do a thorough investigation of any potential quality issues that might be related to various generic products available in the pharmaceutical market.

## Introduction

The global prevalence of hypertension, a chronic public health concern, is predicted to be 1.3 billion at present, and is expected to rise to 1.56 billion by 2030, at an estimated cost of $274 billion [1]. Africa has a high prevalence of hypertension, according to WHO estimates, with 46% of persons 25 years of age and older having high blood pressure [2]. This has grown to be a severe threat to the welfare of people living in sub-Saharan Africa [3–5]. Nonetheless, there is a useful drug that blocks calcium channels, which can lessen the overall effects of hypertension. Amlodipine is a vaso-selective dihydropyridine antihypertensive medication that blocks calcium channels to stop calcium ions from passing through membranes and into cardiac and vascular smooth muscle. It can also be used to treat some arrhythmic disorders, peripheral vascular disease, and angina [6]. Even though there are many different ways to treat hypertension, numerous studies have shown that persons with hypertension lack access to appropriate blood pressure management worldwide, especially in Africa [1, 3, 7]; only 31.0% of people having managed their blood pressure [8].

Study conducted in the regions of Ethiopia have revealed that hypertension is highly prevalent there. For example, in Jimma, the prevalence of hypertension was 21.3% (reported in 2014; 22.2% of males and 19.6% of females) [9]. This was presented as evidence that Jimma Town’s uncontrolled hypertension problem was mostly caused by non-adherence to the prescription regimen. The Comorbidity is prevalent in places like Ethiopia, and these have been found to be the major obstacles to hypertension patients’ adherence to their treatment regimens. On the other hand, it has been demonstrated that a thorough knowledge of the illness increases the chance of medication adherence in this patient group [10]. The report from Jimma Town indicated, the private drug stores, regulatory compliance was not up to standard. Laboratory quality control testing of amoxicillin 500 mg capsules, which revealed evidence of unsatisfactory assay results, and which turn has provided assurance of weak regulatory set-up [11]. Poor treatment outcomes of an antihypertensive drugs may be easier to find within the pharmaceutical market if there is medication non-adherence and weak drug regulatory action, such as ineffective port control, unofficial drug distribution, and weak follow-up of inspections for manufacturers, importers, wholesalers, and retailers. Researchers are interested in evaluating tablets of therapeutically effective antihypertensive pharmaceuticals because of reports of poor antihypertensive tablets, which have been found primarily in Africa and which demand for strict regulation [12].

Despite the lack of vigorous regulatory activity in the pharmaceutical industry, evaluation of the quality of final products and initial supplies are critical to identify the excipients, disintegrants, lubricants, and additives used during formulation as the source of quality problems and the onset of drug effects. The dissolving characteristics of medicinal pharmaceuticals must be assessed in order to determine the rate at which the drugs enter the bloodstream following delivery. Pharmacological substances are categorized into four groups by the Biopharmaceutical Classification System (BCS) according to their solubility and permeability [13]. Amlodipine falls under the BCS Class I, rapidly soluble and highly permeable drug, and granted for Bio waivers according to WHO and European medicine agency guidelines [14].

The belief that pharmaceuticals produced by large companies are better than those made by smaller ones is a widespread psychological fallacy. The test product and reference product must both be subjected to statistical comparison, model dependent, and independent parameters to determine the bioequivalent of the generic products to that of comparator products in order for them to be eligible for a BCS-based bio waiver for BCS Class I drug substances [15]. The problems of poor quality pharmaceuticals are one that is becoming more and more widespread worldwide. Again, it is imperative to ensure that many versions of an active ingredient that are available are pharmaceutically equivalent [16]. Thus, when pharmaceutical products have the same amount of active ingredient in a comparable dose form but may not always contain the same excipients, evaluating pharmaceutical equivalency provides insight into which pharmaceutical items satisfy all applicable quality control standards of similar strength, quality, purity, and potency [17, 18].

The potential of the global pharmaceutical industry to swiftly disseminate substandard medicines across the globe, without sufficient detection and intervention measures in place, is a matter of concern [19]. When it comes to hypertension, using the wrong active component in antihypertensive formulations prevents the wrong active component from suppressing infection. As a result, this opens the door for pathogen growth, underlying disease aggravation, and resistance development [20]. Despite the fact that amlodipine is the most frequently prescribed anti-hypertensive drug, hypertension is the most common non-communicable diseases [21]. Because there is a lack of high-quality post-market monitoring data, it is difficult to make decisions about the efficacy, safety, purity, and bioavailability of Amlodipine medicine in Ethiopia, especially at Jimma Town. Consequently, the study’s objectives were to assess and contrast the bioequivalence and quality control parameters of amlodipine tablets that were sold commercially in Jimma Town drug retail outlets.

### Research questions and hypotheses

In order to accomplish the study’s objective, the three research questions that were proposed were used. (1) Are there any low-quality amlodipine brands on the market? (2) Can the comparator product substituted with all of the sampled amlodipine brands in clinical practice? (3) What is the kinetics of the drug substances’ release from the dosage form?

By answering these three research questions, the study aimed to further understanding of the quality, interchangeability, and release characteristics of amlodipine brands available in the Jimma Pharmaceutical market. When selecting and utilizing amlodipine products, consumers, regulatory agencies, and healthcare professionals can all benefit from the research’ insightful information. The null hypothesis and alternative hypothesis were thought to be associated with the quality control characteristics of various amlodipine brands in order to address the research concerns. The brands of amlodipine in the Jimma Town pharmaceutical market satisfy the necessary requirements for quality control metrics, according to the null hypothesis (H0) while the Jimma Town pharmaceutical market’s amlodipine brands, according to Alternative Hypothesis (Ha), do not adhere to the necessary quality control standards.

## Methodology

### Study setting, and period

The samples were collected from Jimma town pharmaceutical market, Oromia Regional State, South West Ethiopia from July-1 to 30/2019. The experiment of the study was performed at Jimma University in Laboratory of Drug quality control (JuLaDQ) from Aug 30/2019-Feb 27/2020. The town is found 355km away from the capital city of the country (Addis Ababa). The town is the hub of the commercial centre of Southwest Ethiopia with nine (n = 9) legally functioning pharmaceuticals wholesales distributing drugs to 54 drug retail outlets (n = 19, Pharmacies, n = 33, drug stores), health centers (n = 4), clinics (n = 15) and hospitals (n = 2) that delivery health service to a total population of 195 228 people that resides in seventeen (n = 17) kebeles with an estimated 40 450 households [22].

### Chemical/Reagents/Solvents

Amlodipine besylate pure (100%w/w) working standard active pharmaceutical ingredient (API) was obtained (gift) from Ethiopian Food and Drug Authority, High performance Liquid chromatography grade water was prepared in Jimma university laboratory of drug quality (JuLaDQ) using ultra-pure water purification system, Acetonitrile (chromo solve HPLC grade ≥ 99.9% = UN.1648, Lot 52BD06354), Methanol (assay = 99.8%, HPLC grade, lot/batch no. H170751602), Phosphoric acid (assay = 85%, UN 1805, Batch/Lot no. 60091), Tri-ethyl amine (Lot no 44160LR) were used for the study.

### Sampling technique and sample collection

Sampling strategy was designed following the guidelines for field surveys of the quality of medicines proposed by World Health Organization [23]. Accordingly, random sampling techniques employed in the selection of drug outlets. In this regard, the main question focused in the sampling strategy was “Are there medicines of poor quality in Jimma town?” Moreover, the technical aspects of the methods employed in the current study were based on previous study conducted on evaluation of medicine quality. As such, before induction of experimental work the complete list of all available brands of amlodipine tablets and the drug retail outlets selling the amlodipine brands were identified. Accordingly, all available ten brands of the amlodipine tablets with the label claims of 10mg, and 5mg were purchased from pre-identified drug retail outlets of Jimma Town via mystery shoppers thus the risk that the sellers may raise a suspicious and might reduce the required samples were minimized. A total of 1,000 sample tablets were purchased from private and government run pharmacies. One hundred tablets (n = 100) per brand of amlodipine tablets were purchased from the selected drug retail outlets, in their original packaging, as supplied by the manufacturers. During sample collection, all relevant information, including the name of the drug substance, country of origin, manufacturing company, expiry date, manufacturing date, and batch/lot number, was recorded (S1 Table).

One of the tested brands, AMD-1*, was identified as the comparator product, while Amlodipine innovator products weren’t available on the Ethiopian pharmaceutical market. The WHO Guideline on the Selection of Comparator Pharmaceutical Products for Equivalence Assessment of Interchangeable Multisource (Generic) Products, which specifies the selection of a product that has been given approval in an International Conference on Harmonisation (ICH) associated country as a comparator, served as the basis for the choice of the comparator products [24].

For experimental work, all samples were transported to JuLaDQ on the same day, and the samples were kept in their original package as well as stored room temperature until the analysis.

### Quality control parameters

#### Visual inspection

Visual inspection of the physical characteristics of dosage form, packaging and labelling information was conducted according to WHO checklist designed to health professionals to carry out visual inspection of medicines for signs of counterfeiting [25]. All samples underwent visual inspection for brand name, active ingredient name, the number of units per container, the dosage form, country of origin, manufacturing company, strength of the active ingredient, batch/lot number, manufacturing date, expiration date, precaution, storage condition and presence of leaflets/package insert. Then, the physical characteristics of tablets incorporating uniformity of color and size, shapes, breaks, crack, splits or pin holes were visually inspected.

### Friability test

Twenty tablets from each brand of amlodipine were selected randomly, and carefully dedusted before testing. Then accurately weighed and placed in the drum of the friability tester, and rotated at 100 revolutions (25rpm for 4 minute) as per USP friability test guidelines. Remove any loose dust from the tablets as before, and again accurately weighed. Then, the percentage of weight loss was calculated using Eq (1), and compared with United State Pharmacopoeia43-NF38 acceptance limits. The Pharmacopoeia recommended that the percentage loss not more than 1% is considered to be acceptable and if cracked, cleaved, or broken tablets are present within the tablet sample after tumbling, the tablet fails the test [26].


$$\%weight\;loss=\frac{Initial\;weight-Final\;weight}{Initial\;weight}\times 100$$ (1)

#### Weight variation

Twenty tablets (n = 20) randomly selected from each brand of amlodipine and conducted as per United State Pharmacopoeia. Individual tablets were weighed using a calibrated analytical balance. The percentage weight variations of the tablets were calculated using Eq (2). Tablets pass the United States pharmacopoeia standard if not more than (NMT) two tablets were outside the percentage limit, and if no tablets differ by more than two times the percentage limit [27].


$$\%Weight\;variation=\frac{Individual\;weight-Average\;weight}{Average\;weight}\times 100$$ (2)

### Assay study of active ingredients

#### Identification test

The identification test was carried out by comparing the retention time of the peak of Amlodipine in the drug sample under assay test with that of the Working standard [27].

#### Preparation of Buffer and mobile phase

The mobile phase consisted of a 35:15:50 (%v/v) ratio of methanol, acetonitrile, and buffer solution, which was produced by adding 7.0 mL of triethylamine to a 1000 mL flask with 900 mL of water. Then the pH of the buffer solution was adjusted to 3.0±0.1 by Phosphoric Acid, and diluted well to the required volume. Considering USP 36 amlodipine’s official monograph, diluted with water to volume, and thoroughly blended [27].

#### Preparation of standard solution

A 100 ml volumetric flask containing 2 mg of USP amlodipine working standard was used to generate the working standard for an amlodipine solution. Following the addition of mobile phase up to the mark, a solution with a known concentration of 0.02 mg of amlodipine per ml was obtained by sonicating the mixture.

#### Sample solution preparation

Five tablets (n = 5) were picked at random from each brand and placed in a volumetric flask of 500 ml. The mobile phase was then added, and the flask was spun to break up the tablets. The flask was then filled with 300 ml of the mobile phase, sealed with the stopper, and shaken vigorously for 30 minutes. The mobile phase was increased to the volume and well mixed for further dilution. Following that, a sample solution containing 0.02 mg/mL of amlodipine equivalent was produced and passed through a 0.45*μm* pore size syringe tip filter.

#### Chromatographic system

The assay of the amlodipine tablets required the adjustment of the chromatographic conditions. The percentage of active pharmaceutical ingredient was determined using HPLC (Drawell Scientific Agilent) type (Model: DW-LC1620A Binary Gradient System), flow rate of 1 ml/min, column type: C18, column temperature of 30 ± 1°C, and 237 nm detector wavelength. After that, 50*μ*l of the amlodipine working standard concentration was injected into an Agilent HPLC to analyse the retention time, resolution, theoretical plate count, tailing factor, and relative standard deviation.

### Dissolution test study

#### Preparation of dissolution media

The dissolution medium was filled with 500 mL of 0.01N hydrochloric acid (HCl). To generate 0.01 N HCl, 0.9 mL of 37% HCl was dissolved in 1000 mL of distilled water [27].

#### Calibration curve construction

About 14 mg of the powdered working standard were dissolved in 100 ml in a volumetric flask to produce the amlodipine standard stock solution. After that, 0.01N HCl buffer (pH = 1.2) was added up to the mark to provide a solution with a known concentration of about 0.14 mg/ml. The first 7ml of the standard stock solution were transferred to a 50ml volumetric flask and filled with buffer to the necessary amount in order to prepare a standard solution with a concentration of 0.02 mg/ml. The Food and Drug Administration Office of Regulatory Affairs directed that standard solutions be made from working concentrations between 80% and 120%, with an approximate content of 0.016, 0.018, 0.02, 0.022, and 0.024 mg/ml [28]. Then, using spectrophotometry, the absorbance was calculated at a wavelength of 239 nm and plotted against the five concentration levels. The concentration of the working standard for amlodipine was then plotted against absorbance to determine the calibration curve. The equation for the calibration curve of the working standard for amlodipine was depicted in Eq (3):-

Y=mx+b (3)

Where;

Y = Absorbance reading of Amlodipine working standard, m = Slope of the straight line, b = intercept on y-axis, and x = concentration of analyte.

#### Dissolution test procedure

Dissolution testing of amlodipine tablets was conducted according to the United States Pharmacopoeia protocol. Teflon was used to cover the paddle of the USP Dissolution Test Apparatus II in order to shield test samples from stainless steel, which could cause amlodipine tablets to break down. The temperature was held at 37±0.5°C for 45 minutes while the paddle was turned at 75 revolutions per minute (rpm). Then, the dissolution medium was filled with 500ml of 0.01N HCL with a pH of 1.2. Amlodipine tablets (n = 6) of each brand was distributed among the six dissolution vessels after being randomly selected. To maintain sink conditions, an aliquot of 5 ml was removed at 5, 15, 30, and 45 minutes. An equal volume of fresh medium was added after each withdrawal. Using the medium as a blank, the extracted samples were filtered using a syringe with a 0.45μm pore size. The quantification process was carried out using the validated UV absorption spectrophotometric method at a wave length of 239 nm. The percentage of the labelled amount of amlodipine was calculated using Eq (4).


Result=(AUAS)×(CSL)×D×(Mr1Mr2)×V×100 (4)

AU = absorbance of the Sample solution

AS = absorbance of the Standard solution

CS = concentration of the Standard solution (mg/ml)

L = label claim (mg/Tablet)

D = dilution factor of the Sample solution

Mr1 = molecular weight of amlodipine, 408.88

Mr2 = molecular weight of amlodipine besylate 567.06

V = volume of Medium, 500 mL

Tolerances: Not less than (NLT) 75% (Q) of the labelled amount of amlodipine (C20H25N2O5Cl) is dissolved [27].

### Statistical analysis

The laboratory investigation’s data were statistically and graphically evaluated using the SPSS version-25, and Microsoft Excel version 2010. Different brands’ friability, weight variation, assay, and dissolution were compared using the one-way ANOVA Tukey test. Differences that were deemed statistically significant when P<0.05. The *in vitro* dissolution profiles of the various brands were compared using model-independent and model-dependent approaches, and post-hoc Dunnett’s test (p<0.05). The release of drug substance from the dosage form was evaluated using the KinetDS software.

### Model independent approaches

Model-independent approaches were taken into consideration by employing fit factors (F1 and F2), Dissolution efficiency (DE), and Mean dissolution time (MDT) to compare the dissolution profiles of the Amlodipine tablets under study.

### Fit factors

The difference factor (f1) and similarity factor (f2) of Amlodipine (AMD-01 to AMD-10) were determined to choose the optimum formulation from the tested brands using Eq (5), and Eq (6). Two dissolution profiles were considered similar and bioequivalent, if f1 is between 0 and 15 and f2 is between 50 and 100 [29].


$$f1=\{[\sum_{t=1}^{n}|Rt-Tt|]⁄\sum_{t=1}^{n}[Rt]\}\times 100$$ (5)

$$f2=50log\left\{\left[\frac{100}{\sqrt{[1+\sum_{t=1}^{n}\left\{Rt-Tt)2/n\right]}}\right]\right\}$$ (6)

Where;

n = is the number of time points, Rt = is the dissolution value of comparator product at time t, Tt = is the dissolution value for the test product.

### Dissolution efficiency

Dissolution efficiency is the area under the dissolution curve within a time range (t1—t2) [30]. DE was calculated by using the following Eq (7).


$$DE=\frac{\int_{t1}^{t2}y.dt}{y100(t2-t1)X100}$$ (7)

Where;

y = is the percentage dissolved at time t. The integral of the numerator which is the area under the curve was calculated using the following Eq (8).


$$AUC=\sum_{i=1}^{n}(t1-ti-1)(yi-1-yi)/2$$ (8)

Where;

t_i_ = is the i^th^ time point, and y_i_ is the % of dissolved product at time t.

### Mean dissolution time

Mean dissolution time was also considered in the study to characterize the drug release rate from the dosage form and the retarding efficiency of the polymer [31], and calculated using Eq (9).


MDT=∑[ti.ΔQi]/Q∞ (9)

Where;

ti, = is an intermediate time of the intervals of sampling time

ΔQi = is the amount of API dissolved in every interval of t”,

Q∞ is the maximum of API dissolved.

### Model dependent approaches

Several mathematical models have been performed in the interpretation of mechanism of Amlodipine drug release from a dosage form that is a vital tool to understand the drug release kinetics of a dosage form (Table 1). The models were evaluated by deciding a parameter based on the percentage of cumulative drug releases vs. time dependent dissolution profiles. The drug release kinetics were determined as per model dependent drug release kinetics guideline [32].

**Table 1:** Mathematical models for comparison of dissolution profiles amlodipine tablets.

Models	Equation
Zero-order kinetic model	Qt = Q0 + K0t
First-order kinetic model	logQt = logQ0−k1t/2.303
Second-order kinetic model	$\frac{1}{Q}=k.t+\frac{1}{Qo}$
Third-order kinetic model	$\sqrt{Q}=k.t+\surd Qo$
Higuchi kinetic model	Qt=Kh×t½
Weibull kinetic model	log[−ln(1−m)]=βlog(t−Ti)−logα
Hixson-Crowell kinetic model	$\sqrt[{3}]{Qo}-\sqrt[{3}]{Qt}=Khc.t$
Korsemeyer-Peppas kinetic model	Mt/M∝ = Ktn

Where;

Qt = is the amount of drug released in time t

Qo = is the initial amount of drug

Mt/M∝ = is the amount of drug released at time t/the amount of drug released at infinite time t”

m = is accumulated fraction of the drug

β = is shape parameter

α = is scale parameter

Ti is location parameter

n is releasing exponent

ko, k_1_, kh, khc, and k is releasing rate constant.

## Results

Ten distinct brands of amlodipine tablets with label strengths of 5 mg and 10 mg were bought from government-run and privately owned pharmacies in Jimma town. One (n = 1) brand was collected from a locally manufactured company, while the other nine (n = 9) brands were made in four other countries (Europe, Malaysia, India, and Europe). At the time of evaluation, every product had not yet reached the end of its shelf life.

### Visual inspection result

The visual inspection result indicated that with exception of brand AMD5 (Table 3), all evaluated products were within WHO visual inspection guideline [25]. During visual examination no defects were observed except that brand AMD5 had no registration number both on its primary and secondary package. No crack cleaved, or broken tablets were observed on the tablet sample after tumbling (S2 Table).

### The friability test results

In the tablet friability test, the maximum and minimum % friability observed were 0.92% (AMD5), and three brands (n = 3) (AMD4, AMD9, and AMD10) all showed a 0.01% weight loss respectively. The friability percentage for the rest of the brands falls within the range of 0.01% and 0.92%. The results of the percentage weight loss of each brand of amlodipine tablets in Table 2 showed that all tested samples of the tablets met the pharmacopoeia specification limit. USP43-NF38 describes that a satisfactory tablet should have a weight loss of not more than 1% after weight loss. The statistical One way-ANOVA revealed that there was no significant difference (P>0.05) between the mean weight loss of examined amlodipine tablets in the study area.

**Table 2:** Friability and weight variation test results.

Codes	% of loss	Weight in mg (Mean±SD)	Maximum	Minimum	Upper Deviation	Lower Deviation	p-value
AMD-1*	0.21	160.72±2.02	164.7	156.4	2.48	2.69	P = 0.001
AMD-2	0.12	412.31±3.75	427.1	409.1	3.59	0.78	
AMD-3	0.08	125.19±2.3	129.2	120.3	3.20	3.91	
AMD-4	0.01	400.26±3.54	406.1	393.1	1.45	1.79	
AMD-5	0.92	448.53±3.99	455.3	440.7	1.51	1.74	
AMD-1	0.07	398.81±4.81	406.7	389.4	1.98	1.98	
AMD-7	0.19	159.55±2.11	165.2	156.6	3.54	1.85	
AMD-8	0.10	205.97±0.99	207.5	204.3	0.75	0.81	
AMD-9	0.01	106.87±2.13	112.4	102.7	5.17	3.90	
AMD-10	0.01	201.59±2.31	207.9	198	3.13	1.78	

### The weight variation test results

According to the standard of United State Pharmacopoeia, a tablet with the strength of 130mg or less than that, the percentage deviation must not be ˃ ±10.0 for the minimum of 18 tablets and ˃±20 for a maximum of 2 tablets, hence the strength of the samples tablet is 5mg and 10mg and its percent deviation lie within the pharmacopoeia specification limit (Table 2). The maximum upper deviation is 5.17(AMD-9) and the minimum lower deviation is 0.78 that of AMD-2, so according to this data all the brands compiled with the USP specification. The sample mean weight of all brands varied significantly (P<0.05), according to the one-way statistical analysis of variance (ANOVA) result at 95% confidence interval (CI).

### Assay study of the tablets

#### Identification test results of Amlodipine tablets

The high-performance liquid chromatography technique verified that all brands matched the identity test’s requirements by confirming that Amlodipine was used as an active pharmaceutical ingredient (API) in all samples that were tested (S1 Fig). However, the samples and the amlodipine working standard had a very small difference in retention time (-0.905 min). This might result from interactions between excipients and the column, variations in the diluent’s pH, or shifts in the room temperature.

#### System suitability of Amlodipine working standard for assay study

Using 50 μl of the Amlodipine working standard as an injection into the HPLC, the system’s applicability was evaluated. Then, by examining the relative standard deviation, resolution, tailing factor (peak symmetry), and theoretical plate, the performance characteristics were assessed. The system was deemed suitable (Table 3) in respect of relative standard deviation (%RSD; 0.26), tailing factor (average; 0.842), and theoretical plate (average; 12345.68) for amlodipine as per USP36/NF31 requirements. Therefore, the chromatographic system was working perfectly for detecting the respective sample signals.

**Table 3:** Performance parameters test results under the study.

Injection Working Standard(n = 6)	Resolution	Theoretical plate count	Relative standard deviation	Tailing factor
	12.25	12345.68	0.26	0.842
USP specification	NMT8.5	NLT 2500	NMT 1.5%	NMT 2.0
Compliance	Complaint	Complaint	Complaint	Complaint

NB: NMT; Not More Than, NLT; Not Less Than

### Assay test results

All ten (n = 10) distinct brands’ assay results were within the USP standard parameters established by the USP 36 amlodipine official monograph (Table 4). Amlodipine tablets must contain between 90% and 110% of the amlodipine specified on the label, under the pharmacopoeia. AMD-3 (91.27%±1.09) had the least amount of pharmaceutical content compared to other brands, while AMD-8 (102.41%±0.69) had the highest percentage of active substance. The average amount of active ingredients in tablets of the amlodipine brand tested at the study location varied significantly (P< 0.05), according to one-way ANOVA statistics.

**Table 4:** Assay result of tested Amlodipine brand tablets.

Sample	Mean±SD	USP 36 specification	Remarks	p-value
AMD-1*	92.39±2.7	90%-110%	✓	P = 0.001
AMD-2	92.42±2.55		✓	
AMD-3	91.27±1.09		✓	
AMD-4	97.84±1.68		✓	
AMD-5	97.55±2.26		✓	
AMD-6	100±1.95		✓	
AMD-7	99.07±2.35		✓	
AMD-8	102.41±0.69		✓	
AMD-9	99.44±0.72		✓	
AMD-10	95.02±0.95		✓	

### Dissolution study

#### Calibration curve for dissolution study

**Fig 1:**
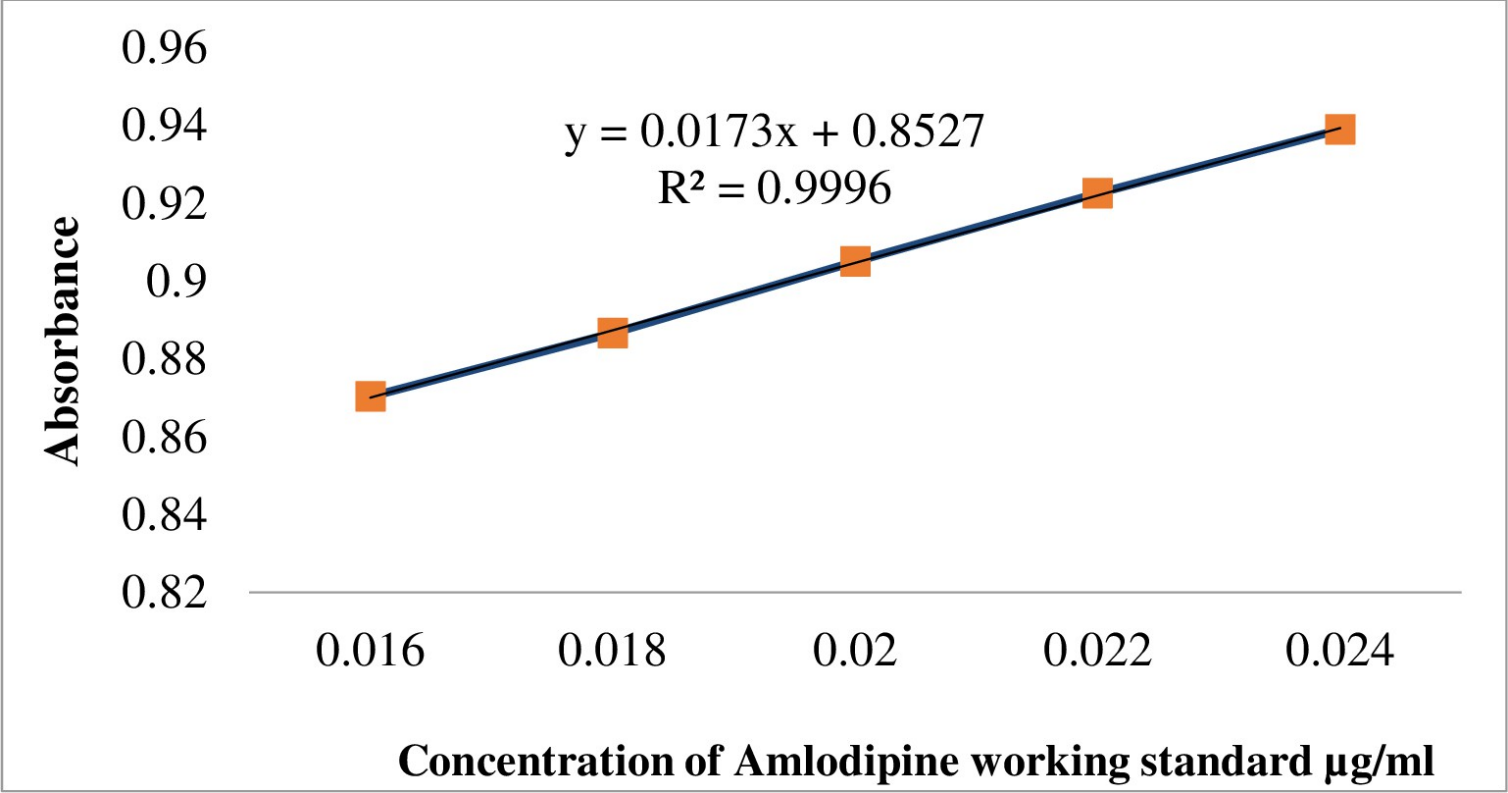
Calibration curve of Amlodipine working standard.

A linear regression equation is y = 0.0173x+0.8527, as shown in S1 Fig, where y is the absorbance and “x” is the concentration of the working standard. Over the concentration range of 0.016 mg/ml to 0.024 mg/ml, the curve demonstrated a strong linear connection between the concentration of the tested samples and the absorbance values (r^2^ = 0.9996) (Fig 1).

### Dissolution test results

**Fig 2:**
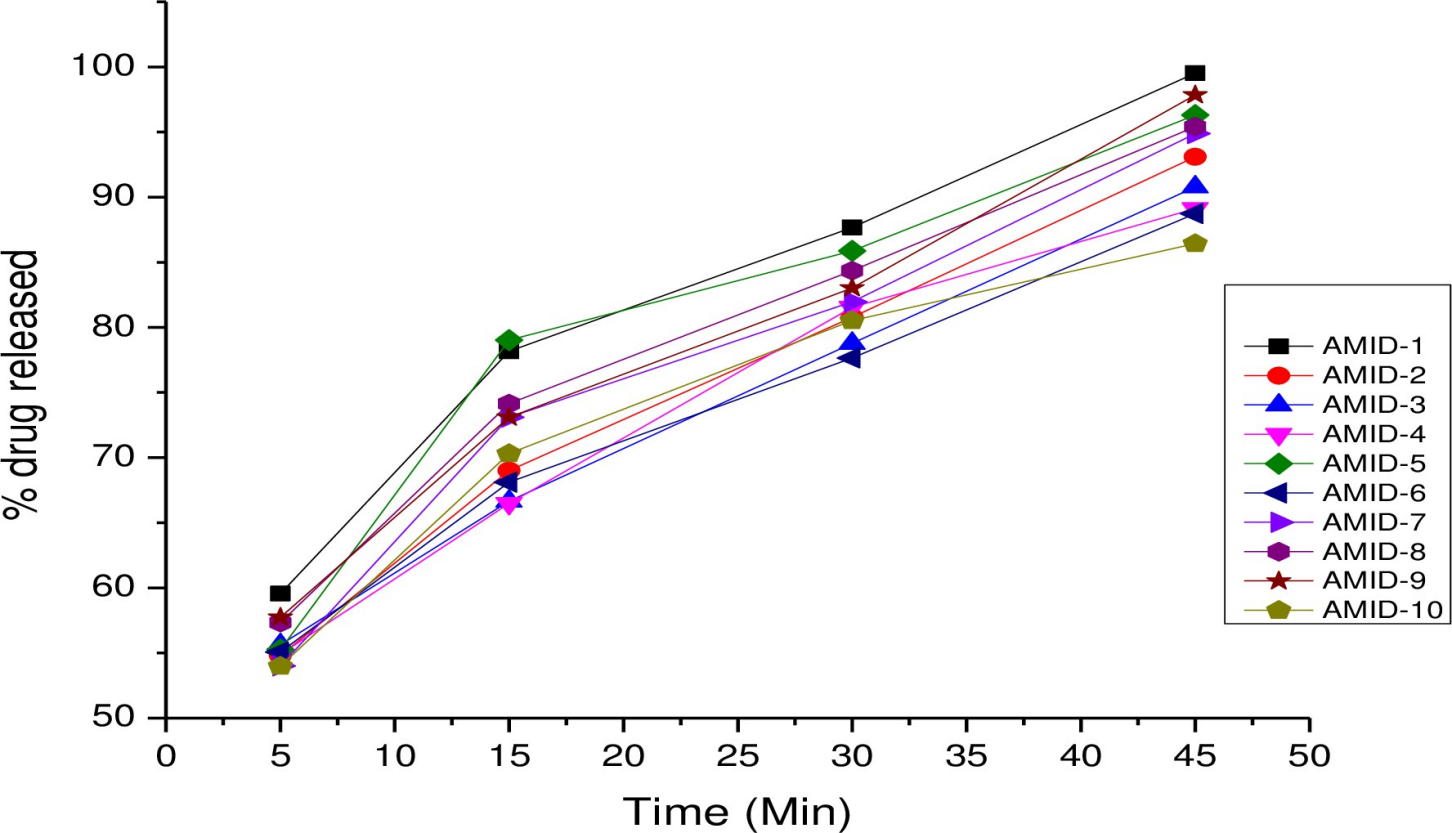
Time dependent dissolution profile results (n = 4).

According to the USP 36 official monograph, amlodipine should release more than 75% (Q) of the indicated amount at a single time point of 30 minutes. All 10 brands of amlodipine tablets released more than 75% of their pharmaceutically active components in less than 30 minutes, as indicated in Fig 2. Before 30 minutes, AMD-1 released 78.17% of its content. AMD-8, AMD-7, and AMD-10 then released 74.15%, 73.09%, and 70.31% of their content, respectively, at the time of 15 minutes. After 30 minutes, all of the remaining brands released ≥75%. These demonstrate that all test brands met the pharmacopoeia tolerance limit outlined in the official United State Pharmacopoeia-36 amlodipine monograph. The dissolution profile comparison, graphical method was undertaken to confirm batch to batch consistency to aware their bio-availability of the distinct product of amlodipine as can be presented in S2 Fig.

### Model independent dissolution profile comparison

In the study, variation in the dissolution profile was found at various time points. Valid statistical one-way ANOVA test employed (p<0.05) using post-hoc Dunnett’s test for further back up the variation in the dissolution profiles of each brand. The outcome demonstrated that there was no difference in their release characteristics between the tested samples and the comparator product (P = 0.9985) (Table 5). This implies the existence of amlodipine compounds with statistically comparable in vitro release profiles.

**Table 5:** Dunnett multiple comparisons test for dissolution profile of Amlodipine tablets tested with comparator.

**(I) brands**	**(J) brands**	**Mean Difference (I-J)**	**95% Confidence Interval**		**P-value**
			**Lower Bound**	**Upper Bound**	P = 0.9985
AMD-2	AMD-1*	2.02750	-30.7712	34.8262	
AMD-3	AMD-1*	0.56500	-32.2337	33.3637	
AMD-4	AMD-1*	0.62250	-32.1762	33.4212	
AMD-5	AMD-1*	6.72250	-26.0762	39.5212	
AMD-6	AMD-1*	7.72000	-25.0787	40.5187	
AMD-7	AMD-1*	3.59500	-29.2037	36.3937	
AMD-8	AMD-1*	5.42000	-27.3787	38.2187	
AMD-9	AMD-1*	5.53500	-27.2637	38.3337	
AMD-10	AMD-1*	0.41750	-32.3812	33.2162	

In order to assure the interchangeability between comparator, and tested samples, a model-independent approach of difference factor (f1) and similarity factor (f2) were utilized. The United State Food and Drug Authority (USFDA) strategy was used with all-time points (n = 4) included in the in vitro dissolution studies to determine the equivalence of all generic amlodipine tablets with the comparator product (AMD-1*) [29]. Model-independent techniques (f1 and f2 factors) were used in the study to analyse ten different amlodipine brands using the AMD-1* brand as a comparator product (Table 6). Since all of the generic amlodipine products in this study had f2 values greater than 50 and f1 values under 15, they could all be used interchangeably with the comparator product. As a result, all brands satisfy the USFDA’s acceptance limit ((f1 value; <15), (f2-value; >50)) based on a comparison of the dissolution profiles’ similarity and bioequivalent.

**Table 6:** Comparisons of dissolution profile by model independent parameters.

Brand code	*f1*	*f2*	DE in %	Difference of DE	MDT
AMD1*	-	-	77.5	0	9.98min
**AMD2**	8.4	57.55	70.7	6.8	1.08min
**AMD3**	10.2	52.84	69.1	8.4	1.07min
**AMD4**	10.1	52.84	69.7	7.8	9.8min
**AMD5**	2.6	76	76	1.5	9.6min
**AMD6**	10.9	52	68.8	8.7	8.7min
**AMD7**	6.46	63.56	72.4	5.1	1.06min
**AMD8**	4.21	72	74.2	3.3	1.00min
**AMD9**	4.07	71.05	74	3.5	1.1min
**AMD10**	10.37	52.43	69.7	7.8	8.67min

**Note:** AMD1

*; comparator product, DE; dissolution efficiency, MDT; mean dissolution time, Difference of Dissolution Efficiency = comparator- tested samples

By measuring the difference of the dissolution efficiency for various brands of amlodipine tablets, the release profiles were also compared in order to ensure the interchangeability of all goods with the comparator product. The study found that all generic brands were comparable to the comparative product since their difference of the dissolution efficiency were under 10% [32]. According to (Table 6), Brand AMD-1 had the highest mean dissolution time (9.98min), while Brand AMD-8 had the lowest (1.00min) mean dissolution time.

### Dissolution kinetics

All of the data from the dissolution test of tablets containing the Amlodipine brand was fitted to a model-dependent kinetics equation. The best fit was determined by the model with the highest correlation coefficient (r2) value. As can be shown in Table 7, the Weibull model (AMD-10), the zero order (AMD-3), and the Korsemeyer-Peppas models were the most effective predictions for the release of the pharmaceutical substance from the dose form. The Korsemeyer-Peppas model (r2 ≥0.9695) was the best descriptive model for determining the amlodipine drug kinetics from the point of view of all brands examined.

**Table 7:** The model dependent dissolution kinetics result of tested Amlodipine brands tablets.

Model dependent parameters		Amlodipine brands code									
Parameters		AMD-1	AMD-2	AMD-3	AMD-4	AMD-5	AMD-6	AMD-7	AMD-8	AMD-9	AMD-10
Zero-order	r^2^	0.9459	0.9831	0.9952	0.9716	0.8752	0.9846	0.9454	0.9568	0.9783	0.9213
First-order	r^2^	0.9080	0.9565	0.9808	0.9487	0.8241	0.9545	0.9043	0.9822	0.9551	0.8737
Second-order	r^2^	0.8622	0.9210	0.9565	0.9214	0.7708	0.9210	0.8535	0.8785	0.9191	0.8315
Third-order	r^2^	0.8128	0.8766	0.9239	0.8876	0.7201	0.8808	0.7990	0.8307	0.8741	0.78801
Higuchi-model	r^2^	-0.5404	-0.2219	-0.5241	-0.4853	-0.3185	-0.8386	-0.1239	-0.5282	-0.3223	-0.9567
Weibull-model	r^2^	0.8528	0.9317	0.9179	0.9602	0.9599	0.9493	0.9395	0.9424	0.8584	0.9988
Hixson-Crowell- model	r^2^	0.9218	0.9675	0.9868	0.9579	0.8416	0.9636	0.91393	0.9346	0.9644	0.8871
Korsemeyer-Peppas model	r^2^	0.9927	0.99037	0.9751	0.9876	0.9695	0.9892	0.9891	0.9958	0.9819	0.9955

The release data were fitted using the well-known empirical equation established by Korsemeyer-Peppas model to explain the dissolving mechanisms from the amlodipine brand tablets (Table 8). To study release kinetics, a graph was plotted between log cumulative % drug release log (Mt /M∞) vs. log time (log t) (S2 Fig).

**Table 8:** Different drug release mechanisms.

Release exponent (n)	Drug transport mechanism	Rate as a function of time
**0.5**	Fickian diffusion	t ^-0.5^
**0.45 to 0.89**	Non -Fickian transport	t ^n-1^
**0.89**	Case II transport	Zero order release
**Higher than 0.89**	Super case II transport	t ^n-1^

Based on the release exponent value of the Korsmeyer-Pepass model, the drug release from the dosage form was calculated. The slope of a graph plotted in the form of log%cumulative drug release vs. log time, which was created from experimental data, was used to calculate the value of the release exponent of “n”. As shown in S2 Fig, the slope of the plot (n = 0.859) was constructed, describing that the release exponent or the diffusion exponent discovered between 0.45 to 0.89, implying that the drug release from the system follow non-Fickian transport [33].

## Discussion

Pharmaceuticals serve as vital to the global health sector’s management of disease, but they must adhere to the proper quality control standards. To have the desired therapeutic effect, drugs must have the necessary physical characteristics and the correct concentration of the active pharmacological ingredient [34]. The most important factors in pharmaceutical setup are quality [35], and therapeutic relevance of medications [36]. Ten amlodipine tablets in total were collected from Jimma town for the purpose of laboratory drug quality control tests. Brand tablets of the medications were gathered, and they were then subjected to quality evaluation tests like friability, weight variation, identification, assay, and dissolution test. Furthermore, for confirming bioequivalence and drug release kinetics, respectively, model independent and model dependent approaches were used.

Proper packaging is essential to protect the pharmaceutical products from physical abrasions (stress) which are risk factors for the product quality defects [37]. The result of the visual inspection of the selected brand tablets (packaging and labelling information) revealed that none of the samples showed signs of falsified except that AMD-5 had no registration number on its primary and secondary package, as defined by the World Health Organization [25]. The friability test is a critical part of process quality assurance, is used to calculate the amount of powder lost from the outer surface of tablets during mechanical and physical handling while being transported [26]. The percent weight loss observed on the current study was fallen within the range of 0.01% to 0.92%. The results of the percentage weight loss of each brand of amlodipine tablets showed that, all tested samples of the tablets met the pharmacopoeia specification limit. USP43-NF38 describes that a satisfactory tablet should have a weight loss of not more than 1% after fibrillation [26]. Similar findings were reported from Nigeria Lagos, and Uyo towns, as well as from Bangladesh, which evaluated brand of amlodipine and found a weight loss of less than 1% [38–40]. These study indicated that, amlodipine products were mechanically, and physically stable. The results of a one-way ANOVA showed that there was no statistically significant difference between the mean weight loss of the amlodipine tablets studied in the study region (P>0.05).

The weight variation test is essential to guaranteeing good manufacturing practises (GMP), optimum tablet size, and uniform formulation content. The United States Pharmacopoeia (USP) stipulates standards for intact dosage unit tablet weight fluctuation. According to the standard of USP, a tablet with the strength of 130mg or less than that, the percentage deviation must not be ˃ ±10.0 for the minimum of 18 tablets and ˃±20 for a maximum of two tablets. The percent deviation was within the pharmacopoeia specification limit. The maximum upper deviation is 5.17(AMD-9) and the minimum lower deviation is 0.78 that of AMD-2, so according to this data all the brands complied with the USP specification [27]. In line with this study, similar findings were detected in Bangladesh [40], Nigeria [39], Pakistan [41]. The sample mean weight of all brands varied significantly (P<0.05) according to the one-way statistical analysis of variance (ANOVA) result at 95% confidence interval (CI). This was taken into account because an appropriate tablet composition and technique had to be established in order for the final dose form to be statistically similar for maintaining the content of tablet uniformity [42].

Achieving pharmaceutical development milestones is essential to moving a given molecule into the clinical practice, but unless the identity of active pharmaceutical ingredients is determined, this doesn’t always translate to successful treatments or better outcomes for patients [43]. Concerning the identification test, the HPLC method confirmed that all brands passed the identity test [27], verifying that all samples had Amlodipine as an active pharmaceutical ingredient (API) in their formulations. However, there was just a slight variance in retention time (-0.905 min) between the samples and the amlodipine working standard. This might be caused by the way excipients interact with the column, the diluent’s pH, or changes in the ambient temperature [44].

Any item or mixture of materials intended to be employed in the manufacturing of a pharmaceutical dosage form and which, when so utilised, forms an active component of that pharmaceutical dosage form are known as active pharmaceutical ingredients [45]. This substance is intended to modify the structure and function of the body or to have pharmacological action or other direct impacts on the diagnosis, treatment, mitigation, or prevention of disease. For a given pharmaceutical product, the amount of active pharmaceutical ingredients present in the dosage form should be optimum for effective treatment success [46]. According to the study, all ten (n = 10) distinct brands’ assay results were within the USP standard parameters established by the USP 36 amlodipine official monograph. According to the pharmacopoeia, amlodipine tablets must contain between 90% and 110% of the amount of amlodipine listed on the label [27]. In comparison to other brands, AMD-8 (102.41%±0.69) had the highest percentage of active ingredient, while AMD-3 (91.27%±1.09) had the lowest proportion of medication content. The study was consistent with the reports from Pakistan [41], India [47], and Bangladesh [48]. However, inconsistent from the study done in Nigeria [49] were all evaluated products not passed assay tests. The average amount of active ingredients in tablets of the amlodipine brand examined at the study location differed significantly (P< 0.05), according to one-way ANOVA statistics. The variability in the active pharmaceutical ingredient’s release from the dosage form was caused by a variety of analytical techniques, including analytical tools, dilution medium like buffer pH, storage conditions, formulation practises, and the quantity of excipients used [50, 51].

Due to the widespread availability of substandard pharmaceutical products in many developing nations [52], it is essential to frequently conduct the necessary tests to efficiently and affordably evaluate bioequivalence (BE). Pharmaceutical product dissolution testing was also employed as a foundation for determining the degree of absorption into the systemic circulation [53]. The discriminatory power is the ability of a test procedure to discriminate between batches manufactured with different critical process parameters and /or critical material attributes which may have an impact on the bioavailability [54]. In vitro dissolution tests are paramount and vital in pharmaceutical set-ups rather than empirical in human in vivo studies, which are not suitable as they are life-threatening since an assumption was initiated in the era of pharmaceutical technology development [55]. The *in-vitro* dissolution test findings should ideally be able to identify all non-bioequivalent batches. According to the United State Pharmacopoeia -36 official monograph, amlodipine should release more than 75% (Q) of the indicated amount at a single time point of 30 minutes. All 10 brands of amlodipine tablets released more than 75% of their pharmaceutically active components in less than 30 minutes, as indicated. Before 30 minutes, AMD-1 released 78.17% of its content. AMD-8, AMD-7, and AMD-10 then released 74.15%, 73.09%, and 70.31% of their content, respectively, at the time of 15 minutes. After 30 minutes, all of the remaining brands discharged ≥ 75% (Q). These demonstrate that all test brands met the pharmacopoeia tolerance limit outlined in the official USP 36 amlodipine monograph.

For comparison of the different brands of amlodipine tablets, statistical approach Dunnett’s test (P<0.05), model independent (*f1*, *and f2*) and model dependent (kinetics of the formulation) were applied on each batch. Based on the high solubility and high permeability, amlodipine tablets falls in class I of Biopharmaceutical Classification System (BCS) [14]. Generic medications are those whose active ingredient’s patent has expired, and they may even incorporate the original product. This indicates that the generics may be marketed as branded goods using the manufacturer’s trade name [56, 57]. The threat of poor drug absorption of substandard products has hampered the trust of generics and the practice of pharmaceutical substitution in Ethiopia. For instance, according to a study conducted by Mekasha YT et al. (2023) in East Shoa town (Adama and Modjo) in Oromia regional state revealed only two brands of generic azithromycin products were deemed better brands (2/6) for interchangeability with comparator products [58]. Valid statistical one-way ANOVA test employing (p<0.05) post-hoc Dunnett’s test was used to further back up the variation in the dissolution profiles of each brand. According to the present finding, demonstrated that there was no difference in their release characteristics between the tested samples and the comparator product (P>0.05). This implies the existence of amlodipine compounds with statistically comparable in vitro release profiles.

The difference factor (f1) and similarity factor (f2) was used to ensure the interchangeability of the comparator and tested samples. The United State Food and Drug Authority technique was employed to compare all generic amlodipine tablets to the comparator product (AMD-1*) at all-time points (n = 4) included in the *in-vitro* dissolving trials [29]. In the study, model-independent methods (*f*_1_ and *f*_2_ factors) were performed for ten brands of amlodipine by using AMD-1* brand as comparator product. Since all of the generic amlodipine products in this study had f2 values greater than 50 and f1 values under 15, they could all be used in place of the comparator product. As a result, all brands satisfy the USFDA’s acceptance limit ((f1 value; 0 and 15), (f2-value; >50)) based on a comparison of the dissolution profiles’ similarity and bioequivalence [26]. The study was different from reports done in Nigeria were only 4/10 were similar to the innovator at pH of 1.2 [49]. This may due to the variation of the pH of dissolution medium used.

In order to assuring the interchangeability of all products with the comparator product, the release profiles were also compared by calculating dissolution efficiency for various brands of amlodipine tablets [59]. According to the study, all the generic brands were similar to the comparator product as the dissolution efficiency was less than 10% [60]. The mean dissolving time derived from the cumulative curves of dissolved API as a function of time was used to characterise the drug release rate from the dosage form and the polymer’s retarding efficiency. In comparison to the other examined brands, the result showed that brand AMD-1 (9.98) had the greatest mean dissolution time and brand AMD-8 (1.00) had the lowest mean dissolution time. Consequently, a prolonged medication release from the dosage form and a delayed onset of action may serve to identify brand AMD-1. While brand AMD-8 has a quicker start of effect and a higher rate of dissolution. The better the polymer’s capacity to hold medications, the higher the MDT value, and vice versa [31].

In the field of pharmaceutical sciences, controlled release formulations are one of a critical manufacturing practice for the exploitation of the modern concept of therapeutic treatment, whose goal is to increase drug effectiveness and patient compliance and reduce administration frequency and side effects related to dosing [61]. This is because of the broad versatility of their applications. Because it entailed in vivo research, the mechanism of drug release from the formulation in the ancient arena was contested [62]. *In-vitro* mathematical modelling serves as a pillar for solving drug release mechanisms very well since, in the best-case scenario, it allows for the prediction of release kinetics prior to the realisation of the release systems [63]. More frequently, it employs model fitting on experimental release data and allows the measurement of some key physical characteristics, such as the drug diffusion coefficient [61, 64]. The current finding revealed that, all of the data from the dissolution test of tablets containing the Amlodipine brand was fitted to a model-dependent kinetics equation. The best fit was determined by the model with the highest correlation coefficient (r2) value [65]. The result of kinetic model studies for amlodipine dissolution profiles at pH 1.2 indicated that the Weibull model (AMD-10), the zero order (AMD-3), and the Korsemeyer-Peppas models were the most effective predictions for the release of the pharmaceutical substance from the dose form. The Korsemeyer-Peppas model (r2 ≥0.9695) was the best descriptive model for determining the amlodipine drug kinetics from the point of view of all brands examined.

To understand the dissolution mechanisms from the amlodipine brand tablets, the release data were fitted using the well-known empirical equation proposed by Korsemeyer-Peppas model. The drug release from the dosage form was determined based on release exponent value (n”) of Korsmeyer-Pepass model. From the experimental data the value of release exponent of n” was determined from the slope of graph ploted in the form of log %cumulative drug release vs. log time. In the study, the slope of the plot was found to be 0.859, which described that the release exponent or the diffusion exponent found between 0.45 and 0.89, which implies that the drug release from the system follows non-Fickian transport [33]. According to the study’s findings, each medicine tested follow multiple dosage form drug release mechanisms.

### Limitation and strenghth of the study

The investigation centers on assessing quality control parameters that influences the efficacy of finalized pharmaceutical products, given the prevalent issue of quality in this sector. Furthermore, the study presents critical information on brand interchangeability in clinical scenarios through in-vitro dissolution testing, eliminating the necessity for live, in vivo studies. Furthermore, the model-dependent results emphasize the relationship between therapeutic ingredients and dose formulations, warning the manufacturing sector of the need of keeping an eye on crucial procedures and material characteristics.

One major drawback of the study was its lack of representativeness for the entire region of Ethiopia. The presence of financial limitations led to biased sampling and technical procedures. In addition, the limitation of the study is not conducting tests at varying pH. The study did not take into account factors like efficacy, safety, or cost-effectiveness. Instead, it solely concentrated on quality control parameters, assuming that the estimated quality products would remain effective and devoid of any harmful effects. Furthermore, important aspects such as pricing, availability, and marketing strategies were not incorporated in the study. From quality control parameters, the hardness and disintegration tests were not performed due to equipment un-availability.

## Conclusion and recommendation

The physicochemical quality control tests performed on all tested amlodipine brand tablets for friability, weight variation, identification, assay, and dissolution were found to be in compliance with the USP. The result of visual inspection of the selected brand tablets (packaging and labeling information) revealed that none of the samples showed signs of falsification except that AMD-5 had no registration number on its primary and secondary packages, as defined by the WHO. All tested brands passed the model-independent parameters, which in turn indicate that they are clinically interchangeable with the comparator products. All the tested products had more than two drug release mechanisms.

However, the dissolution profiles of the products indicated that there was variation in drug releases. The mean dissolution time test showed that brand AMD-1 (9.98 minute) had the greatest mean dissolution time and brand AMD-8 (1.00 minute) had the lowest mean dissolution time. This, in turn, affects the onset of action of the drugs and the rate of dissolution. Additionally, the study noted that, there was a deviation in packaging, and labeling information from the standard. Therefore, properly designed dissolution profiles with varying pH of dissolution medium, and comprehensive visual inspections were strongly advisable for further analyses of the potential issues that might be linked to various generic products throughout the region.

## Supporting information

S1 Table Label information of different brands of 5mg and 10mg Amlodipine tablets. (DOCX)

S2 Table Visual inspection result of tested Amlodipine tablets in study area. (DOCX)

S1 Fig Chromatogram peak of standard, and sample for assay. (DOCX)

S2 Fig Graph of Korsemeyer-Peppas model kinetic release of Amlodipine. (DOCX)
