# Supplementary material for: In vitro comparative quality evaluation of different brands of Amlodipine Tablets Commercially available in Jimma Town, South-western Ethiopia
Source: PLoS One. 2024 Nov 19;19(11):e0310828. doi: 10.1371/journal.pone.0310828 (PMC11575810; doi:10.1371/journal.pone.0310828)
Supplement: S1 Table — (DOCX) [file pone.0310828.s001.docx]

**S1 Table. Label information of different brands of 5mg and 10mg Amlodipine tablets**

| S/N | Brand code | Brand | Country | Batch No | Manufacturing date | Expiry date |
| --- | --- | --- | --- | --- | --- | --- |
| 1 | AMD-1* | Novartis 10mg | Europe | JD4444 | 03/2018 | 03/2021 |
| 2 | AMD-02 | Amlo-Denk-10mg | Germany | 22160 | 01/2019 | 01/2021 |
| 3 | AMD-03 | Amlorine-10mg | Cyprus | 72243 | 05/2017 | 05/2022 |
| 4 | AMD-04 | Amdepin-10mg | India | D19002T272 | O1/2019 | 01/2021 |
| 5 | AMD-05 | Anlodin-10mg | Malaysia | C1002 | 09/2018 | 09/2020 |
| 6 | AMD-06 | Klodip-10mg | India | 55868009 | 10/2018 | 09/2021 |
| 7 | AMD-7 | Amlibon 5mg | Ethiopia | JE1189 | 06/2018 | 05/2021 |
| 8 | AMD-8 | Amlorine 5mg | Cyprus | 73720 | 07/2017 | 07/2021 |
| 9 | AMD-9 | Amdepin 5mg | India | D19005T271 | 05/2019 | 04/2022 |
| 10 | AMD-10 | Amlo-Denk 5mg | Germany | 21857 | 09/2018 | 09/2020 |
