# Supplementary material for: In vitro comparative quality evaluation of different brands of Amlodipine Tablets Commercially available in Jimma Town, South-western Ethiopia
Source: PLoS One. 2024 Nov 19;19(11):e0310828. doi: 10.1371/journal.pone.0310828 (PMC11575810; doi:10.1371/journal.pone.0310828)
Supplement: S2 Table — (DOCX) [file pone.0310828.s002.docx]

| **Packaging, and labelling information** | | | | | | | | | | | | |
| --- | --- | --- | --- | --- | --- | --- | --- | --- | --- | --- | --- | --- |
|  | **Labelling (√=Yes, and x=No)** | | | | | | | | | | | |
| **Sample code** | **Trade/Brand Name** | **Trade/Brand Name** | **Manufacturer’s Name and Logo** | **Manufacturer’s Full Address** | **Medicine Strength (mg/unit)** | **Dosage Form** | **No. of Units per Container** | **Dosage Statement** | **Batch/Lot No.** | **Manufactory and Expiry Date** | **Storage information** | **Leaflet or package insert** |
| AMD-1 |  |  |  |  |  |  |  |  |  |  |  |  |
| AMD-2 |  |  |  |  |  |  |  |  |  |  |  |  |
| AMD-3 |  |  |  |  |  |  |  |  |  |  |  |  |
| AMD-4 |  |  |  |  |  |  |  |  |  |  |  |  |
| AMD-5 |  |  |  |  |  |  |  |  |  |  |  | **x** |
| AMD-6 |  |  |  |  |  |  |  |  |  |  |  |  |
| AMD-7 |  |  |  |  |  |  |  |  |  |  |  |  |
| AMD-8 |  |  |  |  |  |  |  |  |  |  |  |  |
| AMD-9 |  |  |  |  |  |  |  |  |  |  |  |  |
| AMD-10 |  |  |  |  |  |  |  |  |  |  |  |  |

**S2 Table.** Visual inspection result of tested Amlodipine tablets in study area
