# Supplementary material for: In vitro comparative quality evaluation of different brands of Amlodipine Tablets Commercially available in Jimma Town, South-western Ethiopia
Source: PLoS One. 2024 Nov 19;19(11):e0310828. doi: 10.1371/journal.pone.0310828 (PMC11575810; doi:10.1371/journal.pone.0310828)
Supplement: S1 Fig — (DOCX) [file pone.0310828.s003.docx]

**Supplementary File in S1 Fig**

Chromatogram peak of sample (A) and Chromatogram peak of Working standard (B)


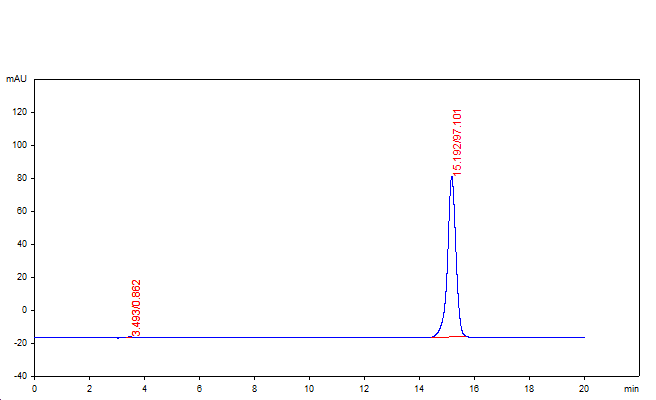


1. Sample peak


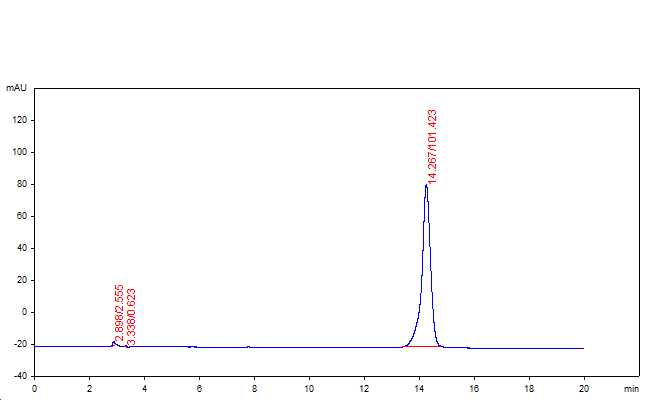


1. Working Standard peak
